# Use of prophylactic oral calcium after total thyroidectomy: a prospective study

**DOI:** 10.1590/2359-3997000000286

**Published:** 2017-09-04

**Authors:** Erwin Langner, Alfio José Tincani, André del Negro

**Affiliations:** 1 Limeira SP Brasil Limeira, SP, Brasil; 2 Universidade Estadual de Campinas SP Brasil Universidade Estadual de Campinas (Unicamp), SP, Brasil

**Keywords:** Calcium, prophylactic, thyroidectomy, study, prospective

## Abstract

**Objective:**

The aim of this study was to evaluate the use of prophylactic oral calcium after total thyroidectomy in the prevention of symptomatic hypocalcemia, and to develop a rational strategy of oral calcium supplementation following this type of surgery.

**Subjects and methods:**

Prospective study including 47 patients undergoing total thyroidectomy from January 2007 to February 2012. The patients were allocated to one of the following groups: I (no postoperative calcium) or II (oral calcium 3 g per day). Oral calcium was started at the first postoperative day and administered until the sixth postoperative day. The patients were followed up for a minimum of 6 months and evaluated with a minimum of five measurements of ionized calcium: preoperative, 16 hours after surgery, seventh postoperative day, and at postoperative days 90 (PO90) and 180 (PO180). The cohort included three men and 44 women, of whom 24 (51.9%) had benign thyroid disease, and 23 had suspected or confirmed malignant disease.

**Results:**

When compared with Group II, Group I had significantly higher rates of postoperative biochemical hypocalcemia at PO1 and PO180, and of symptomatic hypocalcemia at PO1, PO7, and PO90. Other data were not significantly different between the groups.

**Conclusion:**

We conclude that postoperative calcium supplementation effectively prevents symptomatic and biochemical hypocalcemia after total thyroidectomy, and can be safely used after this procedure. The presented strategy of oral calcium supplementation may be implemented in a viable manner.

## INTRODUCTION

The correct execution of any surgical procedure depends on the knowledge of possible complications associated with such procedure. Thyroid surgeries are no exception to this rule. One of the postoperative complications of this type of surgery is hypoparathyroidism, which has a 0.6% to 17% (1) incidence in its permanent form and 1.6% to 87% (2-4) in its transient form. This complication has been a matter of concern to surgeons since the first thyroidectomies have been performed in the contemporary age (5,6).

The proper surgical management of thyroid diseases requires a familiarity with the locoregional anatomy, including the morphology, syntopy, vascularization, and embryology of the thyroid and parathyroid glands. The role of a meticulous surgical technique is well established in the literature, including the dissection of the superior and recurrent laryngeal nerves, careful dissection of the parathyroid glands, and ligation of the peripheral thyroid arteries as the main preventive measures against postoperative complications such as hypoparathyroidism and associated symptoms (1,7). Other causes may contribute to postoperative hypocalcemia, such as surgery extension, surgeon’s experience, resection of one or more of the parathyroid glands, glandular lesions caused by suction in the operatory field and hemodilution (2,8-11), central compartment neck dissection, and reoperations.

In a classical description of the normal parathyroid glands, they are described as varying between one and six in number (a study by Hojaij has described the presence of four glands in 78.56% of 56 patients) (5,12) and as having a kidney-like shape, location in the posterior aspect of the thyroid gland, measurement of 3-8 mm and weight of 15-30 mg, yellow-brownish color, and irrigation by delicate branches of the inferior thyroid artery (1,5,13). Considerable anatomic variants of these glands may be found, and in 1998, Hojaij (5) reported the finding of mediastinal, intrathyroidal, and subcapsular parathyroids in 21.4%, 5.4%, and 14.3% of the cases, respectively.

Unlike temporary hypocalcemia, permanent (or definitive) hypocalcemia lasts for more than 6 months after surgery (3). Both types of hypocalcemia are uncomfortable complications due to their clinical presentation, including the occurrence of the Chvostek’s and Trousseau’s signs, paresthesia, carpopedal spasm, tetany at various levels, electrocardiographic changes, seizures, and behavioral changes. Patients with symptomatic hypoparathyroidism may require prolonged hospitalization, which significantly increases their treatment costs (14). The frequency of postoperative hypocalcemia is significantly greater after total thyroidectomy. In a report of 119,000 thyroid surgeries, Baldassare and cols. (15) found a hypocalcemia rate of 1.9% after partial thyroidectomy and 9% after total thyroidectomy compared with 23.4% after total thyroidectomy and selective bilateral neck dissection.

Several authors (2,16-19) have proposed ways to reduce the occurrence of hypocalcemia, studying predisposing factors and proposing strategies to reduce its incidence and symptoms. The latter includes proposals to prevent hypocalcemia with calcium replacement using calcium carbonate, as reported by Moore in 1994 (20), with effervescent preparations of other types of calcium, as reported by Bellantone and cols. (21), or calcitriol (vitamin D), as described by Bellantone and cols*.* (21) and Tartaglia and cols*.* in 2005 (22). Recently, Docimo and cols. (23) have reported the preoperative and postoperative use of calcium and calcitriol, with a 10% incidence of biochemical hypocalcemia and 6% of symptomatic hypocalcemia when administered for 3 days before and 14 days after surgery.

An analysis of these studies reveals that the preoperative administration of calcium preparations prevents symptomatic hypocalcemia, particularly in its severe forms, with no significant difference between the administration of calcium alone or calcium combined with calcitriol (21).

The objectives of this study were to perform a prospective evaluation of the use of oral calcium supplementation after total thyroidectomy and demonstrate its efficacy in preventing symptomatic hypoparathyroidism, in addition to evaluating a viable strategy for the use of oral calcium supplementation after total thyroidectomy.

## SUBJECTS AND METHODS

This study was performed using data of patients examined and operated on by the same surgical team, coordinated by the main author, in the city of Limeira (São Paulo, Brazil), after approval by the Ethics Committee in Research at all the hospitals and the Ethics Committee in Research at the State University of Campinas (Unicamp) under the protocol number 1014/2010.

A total of 47 patients undergoing total thyroidectomy from January 2007 to August 2012 were studied by sequential analysis. All patients underwent a routine preoperative evaluation that included the measurement of serum electrolytes, cell blood count, coagulation tests, chest X-ray, and an electrocardiogram with evaluation by a cardiology specialist, if necessary.

We measured the patients’ serum ionized calcium and thyroid-stimulating hormone (TSH) levels preoperatively and recorded the following parameters: age, gender, surgery date, prior diagnosis, and presence or absence of a thyroid hormone disorder.

All patients were informed about the procedures in this study by a Statement of Informed Consent, which was signed and approved by the Ethics Committee in Research at the involved institutions. The research was performed with the researchers’ resources.

The exclusion criteria included partial thyroidectomy of any type, partial or total resections of parathyroid tissue, and extended thyroidectomy with neck dissection. One patient was excluded due to laryngeal invasion detected during surgery and was then treated by partial laryngectomy, while another patient was excluded due to the execution of bilateral neck dissection. Overall, 15 patients were lost to follow-up and were excluded from the analysis, and one patient died 30 days after surgery with a diagnosis of thyroid lymphoma.

After surgery, the patients were divided into two groups according to their postoperative treatment:

Group I: 27 patients who did not receive calcium treatment after surgery, except in cases of symptomatic hypocalcemia or detection of ionized calcium below 0.8 mmol/L at 16 hours after surgery.

Group II: 20 patients who received treatment with postoperative oral effervescent calcium (Sandoz FF^®^) 3 g daily for 6 days after surgery.

The patients were evaluated before and after the procedure and were followed up for a minimum of 6 months.

According to the protocol, the patients were followed up with at least five measurements of ionized calcium: before surgery, 16 hours after surgery, on the seventh postoperative day, and 90 and 180 days after surgery.

Administration of oral calcium was maintained in the presence of symptomatic hypocalcemia or persistent serum calcium measurements below 0.8 mmol/L after the sixth postoperative day, until normal calcium measurements were obtained. We considered as hypocalcemia the occurrence of serum calcium levels under 1.1 mmol/L, and as severe hypocalcemia the occurrence of levels below 0.8 mmol/L. Calcium levels between 1.1 and 1.4 mmol/L were considered normal.

The presence and intensity of signs and symptoms of hypocalcemia were recorded in a dedicated form and classified into three groups: absence of symptoms, mild symptoms (paresis or Chvostek’s sign), and severe symptoms (Trousseau’s sign, carpopedal spasm, tetany or cardiac signs and symptoms). The presence and duration of the hypocalcemia after treatment, presence or absence of side effects, and number and characteristics of the parathyroid glands observed during surgery were recorded.

The data of the two groups were analyzed and compared using Fisher’s chi-square test, analysis of variance (ANOVA) for repeated measures, and Wilk’s test using the software SAS, v. 9.2 (SAS Institute, Inc, Cary, NC, USA). The significance level (*p*) was set at 0.05.

## RESULTS

A total of 47 patients were analyzed, including three men (6.4%) and 44 women (93.6%), with a mean age of 52.1 years (standard deviation [SD] 12.8 years, median of 52 years). Total thyroidectomy was performed in 24 patients (51.9%) for treatment of a benign disease and in 23 (48.1%) for treatment of a suspected or previously confirmed malignant disease. Overall, 27 patients (78.7%) presented a normal thyroid function at the time of the surgical indication, six (12.8%) presented hyperthyroidism, and four (8.5%) presented previous hypothyroidism. Data from the two groups are summarized in Table 1.

**Table 1:** General characteristics of the 47 patients included in the study

	n	%
Sex		
F	44	93.6
M	3	6.4
Age		
N	47	
Mean	52.1	
Standard deviation	12.8	
Median	52.0	
Indication		
CPG	18	38.3
MNG	3	6.4
GD	3	6.4
PC	16	34.0
Sca	3	6.4
FT	4	8.5
TSH		
High	4	8.5
Low	2	4.3
Normal	41	87.2
Postop hypocalc		
No	33	70.2
Yes	14	29.8
Hypo/hyperthyroidism		
Hyper	6	12.8
Hypo	4	8.5
Normal	37	78.7

F: female gender; M: male gender; n: sample size; indication: surgery indication; CPG: compressive goiter; MNG: multinodular goiter; GD: Graves’ disease; PC: papillary carcinoma; SCa: clinical or cytological suspicion of cancer; FT: follicular tumor; TSH: previous thyroid stimulating hormone; Postop hypocalc: postoperative hypocalcemia; hypo/hyperthyroidism: previous hypothyroidism or hyperthyroidism.

All patients underwent total thyroidectomy; 33 patients showed no signs and symptoms of hypocalcemia (70.2%) while 14 others (29.8%) presented mild symptoms of hypocalcemia. No severe symptoms of hypocalcemia were observed.

Biochemical hypocalcemia occurred in six patients (12.8%) in the preoperative evaluation, in 23 patients (48.9%) on the first postoperative day, in 17 patients (36.2%) on the seventh postoperative day, in 15 patients (31.9%) 90 days after surgery, and in nine patients (19.2%) 180 days after the procedure, while seven patients (14.9%) still had hypoparathyroidism at the end of this study. Among the six patients with preoperative hypocalcemia, four maintained their status of biochemical hypocalcemia at the first postoperative day (66.6%), two at the seventh postoperative day (33.3%), four at 90 days after the procedure (66.6%), and two cases at 180 days after the surgery (33.3%), while two patients with preoperative hypocalcemia (both in Group I, in which no postoperative calcium was administered) presented all measurements below 1.1 mmol/L). These data are shown in Table 2.

**Table 2:** Postoperative progression of serum calcium levels (mmol/L)

Values (in mmol/L)	n	%
Preop		
0.8 to 1.1	6	12.8
Normal	41	87.2
POi		
0.8 to 1.1	23	48.9
Normal	24	51.1
PO7		
< 0.8	2	4.3
0.8 to 1.1	15	31.9
Normal	30	62.8
PO90		
0.8 to 1.1	15	31.9
Normal	32	68.1
PO180		
< 0.8	3	6.4
0.8 to 1.1	6	12.8
Normal	38	80.8

Preop: preoperative; Poi: immediate postoperative; PO7: 7 days after surgery; PO90: 90 days after surgery; PO180: 180 days after surgery.

The prevalence of biochemical hypocalcemia in both groups and the statistical comparison according to serum calcium level are described in Table 3. The progression of the calcium levels according to the study group and presence or absence of symptomatic hypocalcemia are resumed in Table 4. The correlation between the occurrence of hypocalcemia and symptoms in both groups, evaluated using ANOVA for repeated measures, is shown in Table 5.

**Table 3:** Incidence of postoperative hypocalcemia according to study groups

Values (in mmol/L)	Group				p

	I		II		
Preop					0.2205
0.8 to 1.1	5	18.5%	1	5.0%	
Normal	22	81.5%	19	95.0%	
POi					0.0254*
0.8 to 1.1	17	63.0%	6	30.0%	
Normal	10	37.0%	14	70.0%	
PO7					0.2912
< 0.8	1	3.7%	1	5.0%	
0.8 to 1.1	11	40.7%	4	20.0%	
Normal	15	55.6%	15	75.0%	
PO90					0.1315*
0.8 to 1.1	11	40.7%	4	20.0%	
Normal	16	59.3%	16	80.0%	
PO180					0.0409
< 0.8	2	7.4%	1	5.0%	
0.8 to 1.1	6	22.2%	0	0.0%	
Normal	19	70.4%	19	95.0%	

Fisher’s test / * Chi-square test.

Preop: preoperative; Poi: immediate postoperative; PO7: 7 days after surgery; PO90: 90 days after surgery; PO180: 180 days after surgery.

**Table 4:** Progression of serum calcium levels according to group/symptoms

IV.1 – Progression of serum calcium levels according to groups								
Measurement	Group I (n = 27)			Group II (n = 20)			Time Effect	Group Effect
	
	Mean	Standard deviation	Median	Mean	Standard deviation	Median		
Preop	1.16	0.07	1.20	1.22	0.09	1.22	-	-
POi	1.09	0.09	1.09	1.16	0.08	1.16	< 0.0001	0.0013
PO7	1.10	0.11	1.12	1.17	0.16	1.21	0.0087	0.0028
PO90	1.12	0.12	1.14	1.17	0.14	1.21	0.0448	0.0088
PO180	1.13	0.17	1.14	1.20	0.12	1.23	0.4771	0.0056

**IV.2 – Progression of serum calcium according to symptoms.**								

**Measurement**	**Asymptomatic (n = 33)**			**Symptomatic (n = 14)**			**Time Effect**	**Group Effect**
	
	**Mean**	**Standard deviation**	**Median**	**Mean**	**Standard deviation**	**Median**		

							0.0001	< 0.0001
Preop	1.20	0.09	1.20	1.17	0.08	1.20	-	-
POi	1.16	0.07	1.16	1.04	0.07	1.06	< 0.0001	0.0005
PO7	1.17	0.09	1.18	1.02	0.17	1.05	0.0007	0.0016
PO90	1.18	0.11	1.20	1.05	0.14	1.06	0.0077	0.0022
PO180	1.19	0.12	1.22	1.08	0.19	1.13	0.2490	0.0102

ANOVA for repeated measures. Preop: preoperative; Poi: immediate postoperative; PO7: 7 days after surgery; PO90: 90 days after surgery; PO180: 180 days after surgery.

**Table 5:** Hypocalcemia and presence of symptoms in both study groups

Measurement	Group I, asymptomatic			Group I, symptomatic (n = 10)			Group II, asymptomatic (n = 16)			Group II, symptomatic (n = 4)			p
			
	(n = 17)	SD	Median	Mean	SD	Median	Mean	SD	Median	Mean	SD	Median	
Preop	1.17	0.07	1.19	1.16	0.08	1.20	1.23	0.09	1.24	1.19	0.08	1.18	0.0620
POi	1.14	0.07	1.12	1.02	0.07	1.03	1.18	0.08	1.17	1.08	0.06	1.08	< 0.0001
PO7	1.14	0.06	1.15	1.03	0.15	1.01	1.21	0.10	1.22	1.00	0.26	1.07	0.0009
PO90	1.14	0.11	1.16	1.08	0.12	1.08	1.22	0.08	1.23	0.97	0.17	0.97	0.0007
PO180	1.17	0.16	1.18	1.05	0.16	1.13	1.22	0.05	1.23	1.14	0.27	1.19	0.0678

ANOVA. SD: standard deviation; Preop: preoperative; Poi: immediate postoperative; PO7: 7 days after surgery; PO90: 90 days after surgery; PO180: 180 days after surgery.

## DISCUSSION

Although the parathyroid glands were first described in Indian rhinoceros by Owen in 1852 (cited by Thompson) (26), the relationship between hypocalcemia after total thyroidectomy and resection or injury to the parathyroid glands was only established in 1891, with the first report of tetany after thyroidectomy occurring in 1877 (4). At that same year, Sandström (27,28) started to observe the parathyroid glands in animals and after dissecting 50 human bodies, described in 1880 the anatomy, number, and shape of these glands. Sandström suggested the name of the parathyroid gland while recognizing the independence of its anatomic structure in relation to the thyroid gland. The association between the parathyroid glands and tetany was recognized after 1890 according to Thompson (26) and as cited by Hojaij (5). In 1907, Pool (29) and Hojaij (5) coined the term *tetania paratireopriva*. Also in 1907, Halsted and Evans (30), as well as Reeve and Thompson (31), after dissecting 20 human bodies, confirmed the need for prevention of parathyroid injury during thyroid surgery and identified one delicate arterial bunch for each gland, derived from the inferior thyroid artery in 90% of the patients. Many authors, including Lahey in 1926 (32), Milzner in 1927 (6), Murley and Peters in 1961 (33), and Croyle and Oldroyd em 1978 (34), reported a 10 to 24% incidence of parathyroid resections in thyroidectomy, which led Loré and Pruet in 1983 (35) and Shaha and cols. in 1991 (36) to suggest a meticulous examination of the surgical specimen for identification of parathyroid glands potentially removed during surgery, with the intention of surgically reimplanting them.

Careful dissection, preservation of the parathyroid glands, and peripheral vascular ligation of the thyroid arteries with minimum damage to the parathyroid irrigation, associated with preservation and eventual parathyroid reimplantation, remain today as time-honored surgical procedures to prevent hypoparathyroidism after thyroid surgery.

The prevalence of female patients in this study, as well as their mean and median age, are consistent with data found in the literature and caused by the larger prevalence of thyroid disorders in female patients when compared with male ones.

Some authors (37-39) correlate the decrease in serum calcium levels at the first postoperative day as a prognostic factor of the occurrence of postoperative hypocalcemia after total thyroidectomy. This correlation is more precise with measurement of ionized calcium, which may be safely used for research in hypoparathyroidism (18) since ionized calcium is not affected by variations in protein concentrations as occurring with total calcium.

Other factors identified as responsible for decreasing calcium levels after total thyroidectomy include intraoperative hemodilution, which explains the occurrence of hypocalcemia in other extracervical surgeries with similar extension (2,8), and the hungry bone syndrome, in which a normal parathyroid function is maintained (9). Clark and Duh (40) suggested in 1989 that the parathyroid glands located above the thyroid gland have a higher risk of intraoperative injury due to the longer extension of their vascular pedicles, which have to be dissected during the procedure.

In this study, the first postoperative calcium measurement was performed at 16 hours after the surgery. This decision followed the findings by Bentrem and cols. (18) in 2001, who reported a 94.5% ability to predict postoperative hypocalcemia when calcium is measured at this time point. Marohn and LaCivita (17) in 1995 measured serum calcium levels at 8, 14, and 20 hours after thyroidectomy and concluded that the levels decrease in most cases, reaching their lowest at 14 hours after surgery.

In the present study, biochemical hypocalcemia was observed in the first postoperative day in 48.9% of the patients, reaching 63% in patients in Group I and 30% in those in Group II (*p* = 0.0254) (Table 3). A significant difference in calcium measurement was observed between the two groups 180 days after surgery; of nine patients with hypocalcemia (19.2%), eight belonged to Group I and one to Group II (*p* = 0.0409). The incidence of permanent hypocalcemia (14.9%) was consistent with that in the literature (17%) (1). Other measurements (preoperative [PO] 7, PO90) showed no statistical differences between the study groups, with rates of hypocalcemia of 12.8% before surgery, 36.2% at 7 days after surgery, and 31.9% at 90 days after the procedure, as reported in Table 2.

The incidence of preoperative hypocalcemia (12.8%) suggested a need for routine measurements of calcium, although the possibility of measuring serum calcium levels is not always available in the preoperative protocols in services performing thyroidectomy among us. The progression of patients with preoperative hypocalcemia, with incidence greater than the mean in the PO1, PO90, and PO180, suggests an ability to predict hypoparathyroidism after total thyroidectomy.

The incidence of postoperative biochemical hypocalcemia was 63% on the first postoperative day without the use of prophylactic calcium, which is consistent with data from the literature (2,4,19,24).

Patients in Group II received 3 g of oral calcium daily for a minimum of 6 days, following the procedure reported by Bellantone and cols. (21) in 2002. These authors measured the levels of serum calcium on the first, second, third, and seventh postoperative days, and reported a significant decrease in biochemical hypocalcemia at PO2 and PO3 and a significant decrease in symptomatic hypocalcemia in all study groups.

Some authors (37) have associated the occurrence of symptomatic compressive goiter to a significant risk of postoperative hypocalcemia, while others have reported that the risk of hypocalcemia is greater after surgery performed for malignant tumors (15,41). Dedivitis and cols. (24) observed in a prospective study no significant difference in postoperative hypocalcemia according to the indication for thyroidectomy. Similarly, the present study found no significant correlation between surgical indication and the incidence of postoperative hypocalcemia.

We observed no significant differences in the postoperative progression of calcium levels according to gender, due to the low number of male patients, although the data suggest the occurrence of a higher calcemia in men.

The prevalence of symptoms was clearly related to the occurrence of low serum calcium levels, which is consistent with the calcium physiology. A significant greater rate of symptomatic hypocalcemia was observed in patients who did not receive calcium at the PO1, PO7, and PO90, while in other measurements we observed no significant differences, as shown in Table 5. The data suggest an effective prevention of symptomatic hypocalcemia with the use of oral calcium, which leads to safe and early hospital discharge, according to the literature on this topic (20-22).

Outpatient thyroid surgery with same-day discharge is still avoided by the majority of the authors (42). Lo Gerfo (42) in 1998 defended the implementation of surgery on an outpatient basis, while Clark and Ituarte (42) opposed to it. Schwartz (42) concluded that outpatient surgery yields no financial benefit to the patients, despite reducing costs in 13-30%. A discharge on the first postoperative day, if associated with the prevention of hypoparathyroidism, is considered safe by various authors (42,43), and with marked reduction of costs, an advantage due to more efficient techniques of prevention and control of bleeding, pain, and postoperative hypocalcemia, resulting in a 32 to 56% reduction in hospital-associated costs (44).

In a Colombian report, Sanabria and cols. (45) studied the use of prophylactic calcium and vitamin D, analyzing its cost-benefit and reporting its effectiveness. The total cost of the treatment remains below US$ 2 a day, while calcium measurements due to symptomatic hypocalcemia cost US$ 3.86 and the additional hospital daily fee costs US$ 33.12.

In this report, we observed a significant difference in the incidence of hypocalcemia between the study groups after 180 days of surgery, suggesting the efficacy of the use of prophylactic oral calcium in the prevention of permanent hypoparathyroidism. In contrast, Pattou and cols. (2) in 1998 showed that low calcium levels have a high predictive value of hypoparathyroidism in patients not receiving calcium after thyroidectomy.

In the present study, all patients were operated on by the same surgical team in Limeira (São Paulo, Brazil), and coordinated by the main author. They all underwent standard thyroidectomy with careful dissection of the parathyroid glands and peripheral ligation of the thyroid arteries near the thyroid capsule, which prevents hypoparathyroidism, as demonstrated by Thomusch and cols. (7) in an extensive multivariate analysis with 5846 consecutive patients.

In conclusion, in this study, surgical indication showed no relationship with the incidence of postoperative hypocalcemia. We observed a lower incidence of permanent hypoparathyroidism after the use of the suggested regimen, prediction of postoperative hypocalcemia with early measurement of postoperative calcium, prediction of hypocalcemia after total thyroidectomy with preoperative calcium measurement, and ability to discharge the patient early and safely with a minimum of 24-hour of hospitalization.

A careful surgical technique, including peripheral ligation of the thyroid arteries and meticulous dissection of the parathyroid glands, remains the best approach to preventing hypocalcemia after total thyroidectomy.

The use of prophylactic oral calcium after total thyroidectomy significantly reduced the incidence of laboratory and symptomatic hypocalcemia and may be implemented in a simple, efficient, and safe manner. The strategy shown in this study may be reproduced in a viable and rational way.
